# Plant-Parasitic Nematodes and their Effects on Ornamental Plants: A Review

**DOI:** 10.2478/jofnem-2023-0007

**Published:** 2023-04-14

**Authors:** Amanda D. Howland, Marisol Quintanilla

**Affiliations:** Michigan State University, Department of Entomology, East Lansing, MI 48824 US

**Keywords:** floriculture industry, management, ornamental plants, perennials, plant-parasitic nematodes, symptoms

## Abstract

Worldwide, the ornamental plant industry is estimated to be valued at $70 billion, with the United States’ ornamental plant industry valued at $4.8 billion in 2020. Ornamental plants are cultivated for numerous reasons worldwide, such as decorative, medicinal, social, and utility purposes, making the ornamental field a high growth industry. One of the main pathogen groups affecting the yield and growth of the ornamental plant industry is plant-parasitic nematodes, which are microscopic roundworms that feed on plant parts causing significant yield loss. There are many kinds of plant-parasitic nematodes that affect ornamental plants, with the main genera being *Meloidogyne* spp., *Aphelenchoides* spp., *Paratylenchus* spp., *Pratylenchus* spp., *Helicotylenchus* spp., *Radopholus* spp., *Xiphinema* spp., *Trichodorus* spp., *Paratrichodorus* spp.*, Rotylenchulus* spp., and *Longidorus* spp. The aim of this review is to focus on the effects, hosts, and symptoms of these major plant-parasitic nematodes on ornamental plants and synthesize current management strategies in the ornamental plant industry.

Plant-parasitic nematodes (PPNs) are a global pathogen and are estimated to cause $173 billion in economic loss to agriculture worldwide, with over $13 billion in annual agricultural loss in the United States Elling, 2013). Plant-parasitic nematodes are known to infect every species of cultivated plants and a large majority of weeds, making them one of the greatest threats to crops worldwide Handoo, 1998). These pests are microscopic, aquatic roundworms that live in the soil or in plant parts, and feed on plant tissues causing significant yield loss. The damage caused by nematodes can be extensive and are a result of the nematodes using their stylet, a hypodermic-like mouthpart, to puncture plant cells, inject enzymes and hormones, and remove cell contents when feeding. Due to their feeding, diseased plants are generally stunted, chlorotic, wilted, and have reduced and deformed root systems. This leads to reduced yields, reduced winter hardiness, dieback in perennials, and predisposition of the plant to secondary infections by other pathogens Anwar and Van Gundy, 1989; Handoo, 1998; Phani et al., 2021). Roughly 5 to 10% of all crop losses worldwide are due to PPNs Mitiku, 2018).

While much research has been devoted to the effect and management of PPNs in agricultural crops, one of the less-studied fields is their effect on the ornamental plant industry. This is a huge oversight, considering that the worldwide ornamental plant industry has an estimated value of $70 billion Madhavan et al., 2021). The ornamental plant industry is a high demand, high growth industry, and is a valuable export to many countries Madhavan et al., 2021). The countries with the highest ornamental production are the Netherlands, China, and the United States Darras, 2020), with developed countries being the dominant consumers Adebayo et al., 2020). Cut flowers are one of the top commodities, along with flower bulbs, cut foliage, and bedding plants Adebayo et al., 2020; Darras, 2020).

In the United States, the floriculture industry was valued at $4.8 billion in 2020 (USDA, 2021) and the environmental horticulture industry, or green industry, which is comprised of nurseries, greenhouses, turfgrass sod producers, landscape design, construction and maintenance firms, and wholesale and retail distribution centers, was valued at $159.6 billion in 2018 Hall et al., 2020). The United States’ largest producers in the ornamental plant industry are Florida, California, Michigan, New Jersey, and Ohio. Florida is by far the largest contributor to the U.S. ornamental plant industry, with a value of $1.14 billion (USDA, 2021). Within the United States’ ornamental industry, the highest category of sales went to annual bedding, followed by potted flowering plants, foliage plants, herbaceous perennial plants, propagative materials, and then cut plants and flowers (USDA, 2021). One of the fastest growing aspects in the North American ornamental plant industry is specialty cut flower production due to a huge consumer demand Darras, 2020).

Some of the main ways plants are grown in the ornamental plant industry is by tissue culturing and propagation of corms, plugs, bulbs, and rhizomes. Plant-parasitic nematodes, pathogens, diseases, viruses, and insects can be easily spread through this propagation of plant parts. One of the most important pathogens on this list are PPNs. Within the ornamental plant industry, there are many nematodes that affect ornamental plants worldwide Table 1), with the main genera being *Meloidogyne* spp., *Aphelenchoides* spp., *Paratylenchus* spp., *Pratylenchus* spp., *Helicotylenchus* spp., *Radopholus* spp., *Xiphinema* spp., *Trichodorus* spp., *Paratrichodorus* spp.*, Rotylenchulus* spp., and *Longidorus* spp. Taylor, 1972; Bala and Hosein, 1996; Handoo, 1998; Inserra et al., 1998; Mitiku, 2018). In this review, the most important PPNs and their effects on ornamental crops will be focused on, along with their current and future management strategies.

**Table 1:** Plant-parasitic nematodes found in floricultural crops worldwide and their referenced papers.

Host Scientific Name	Host Common Name	Nematode Species	Reference
*Hippeastrum* sp.	Amaryllis	*Pratylenchus coffeae* (Zimmerman, 1898) Filipjev and Schuurmans Stekhovem, 1941; *P. hippeastri* Inserra, 2007	Crow and Duncan, 2018; Inserra et al., 2007; Pinochet and Duarte, 1986
*Anthurium* sp.	Anthurium	*Aphelenchoides fragariae*; *A. ritzemabosi*; *Helicotylenchus dihystera* (Cobb, 1893) Sher, 1961; *Meloidogyne incognita*; *M. javanica*; *Paratylenchus minutus* Linford in Linford, Oliveira and Ishii, 1949; *P. tenseness*; *Pratylenchus coffeae*; *Radopholus similis*; *Rotylenchulus reniformis*	Bala and Hosein, 1996; Kohl, 2011; Phani et al., 2021; Sipes and Myers, 2018; Wang et al., 2013
*Didiscus caeruleus*	Blue lace flower	*Meloidogyne* spp.	Wang and McSorley, 2005
*Buxus* sp.	Boxwood	*Meloidogyne incognita*; *Mesocriconema* spp.; *Pratylenchus* spp.; *P. vulnus* Allen and Jensen, 1951; *Rotylenchus buxophilus*	Eisenback, 2018; Lehman, 1984
*Caladium* sp.	Caladium	*Meloidogyne* spp.	Gu et al., 2022
*Calendula officinalis*	Calendula	*Aphelenchoides ritzemabosi*; *Meloidogyne* spp.	Kohl, 2011; Wheeler et al., 2018
*Dianthus caryophyllus*	Carnation	*Criconema xenoplex* (Raski, 1952) Loof and de Grisse, 1967; *Ditylenchus myceliophagus* Goodey, 1958; *Helicotylenchus digonicus* Perry, 1959; *H. dihystera*; *H. pseudorobustus* (Steiner, 1914) Golden, 1956; *Heterodera daverti* Wouts and Sturhan, 1979; *Longidorus elongatus* Micoletzky, 1922; *Meloidogyne incognita*; *M. javanica*; *Pratylenchus neglectus* (Rensch, 1924) Filipjev and Schuurmans-Stekhoven 1942; *P. thornei* Sher and Allen, 1953; *Rotylenchulus reniformis*; *Xiphinema diversicaudatum* (Micoletzky, 1927), Thorne, 1939	Borgohain, 2016; Chandel et al., 2010; Deimi et al., 2008; Lung et al., 1997; Phani et al., 2021; Taylor, 1972
*Chrysanthemum* sp.	Chrysanthemum	*Aphelenchoides besseyi*; *A. fragariae*; *A. ritzemabosi*; *Ditylenchus myceliophagus*; *Helicotylenchus digonicus*; *H. pseudorobustus*; *H. vulgaris* Yuen, 1964; *Meloidogyne incognita*; *M. javanica*; *Pratylenchus neglectus*; *P. penetrans* (Cobb, 1917) Filipjev and Schuurmans-Stekhoven, 1941; *P. thornei*; *Rotylenchulus reniformis*	Borgohain, 2016; Christie and Birchfield, 1958; Deimi et al., 2008; Handoo, 1998; Khan, 2015; Kohl, 2011; Mitiku, 2018; Yamamoto and Toida, 1995
*Dahlia* sp.	Dahlia	*Aphelenchoides ritzemabosi*	Khan, 2015
*Hemerocallis* sp.	Daylily	*Aphelenchoides ritzemabosi*; *Helicotylenchus dihystera*; *Meloidogyne arenaria*; *M. hapla*; *M. incognita*; *Paratrichodorus* spp.; *Paratylenchus* spp.; *Pratylenchus* spp.; *Rotylenchulus reniformis*; *Scutellonema brachyurus* Steiner, 1938	Howland et al., 2022; Inserra et al., 1995; Kohl, 2011; LaMondia, 1996; Ye, 2018
*Xanthosoma* sp.	Elephant’s ears	*Meloidogyne* spp.; *Rotylenchulus reniformis*; *Pratylenchus coffeae*	Jatala and Bridge, 1990
*Gardenia jasminoides*	Gardenia	*Aphelenchoides fragariae*; *Meloidogyne* spp.	Crow and Duncan, 2018; Kohl, 2011
*Geranium* sp.	Geranium	*Aphelenchoides fragariae*	Kohl, 2011
*Gerbera jamesonii*	Gerbera	*Aphelenchoides fragariae, Longidorus elongatus, Meloidogyne incognita, M. javanica, Pratylenchus coffeae, Rotylenchulus reniformis*	Borgohain, 2016; Kohl, 2011; Phani et al., 2021
*Alpinia* sp.	Ginger lily	*Criconemella onoensis* (Luc, 1959) Luc and Raski, 1981; *Helicotylenchus dihystera*; *H. pseudorobustus*; *Meloidogyne incognita*; *Peltamigratus* spp.; *Pratylenchus* spp.; *Rotylenchulus reniformis*; *Tylenchorhynchus annulatus* (Cassidy, 1930) Golden, 1970	Bala and Hosein, 1996
*Gladiolus grandiflorus*	Gladiolus	*Aphelenchoides besseyi*; *Ditylenchus myceliophagus*; *Helicotylenchus crenacauda* Sher, 1966; *H. digonicus*; *H. pseudorobustus*; *Meloidogyne incognita*; *Pratylenchus coffeae*; *P. thornei*; *Xiphinema americanum* Cobb, 1913	Borgohain, 2016; Deimi et al., 2008; Taylor, 1972
*Alcea rosea*	Hollyhock	*Meloidogyne incognita*	Khan et al., 2005; Wheeler et al., 2018
*Hosta* sp.	Hosta	*Aphelenchoides* spp., *A. fragariae*	Jagdale and Grewal, 2006; Kohl, 2011
*Hydrangea macrophylla*	Hydrangea	*Aphelenchoides besseyi, A. fragariae, Ditylenchus dipsaci*	Kohl, 2011; Ye, 2018
*Iris* sp.	Iris	*Aphelenchoides fragariae, A. ritzemabosi, Helicotylenchus digonicus, H. pseudorobustus, H. vulgaris, Pratylenchus neglectus, P. thornei*	Deimi et al., 2008; Kohl, 2011
*Ipomoea purpurea*	Morning Glory	*Aphelenchoides ritzemabosi, Meloidogyne* spp.	Kohl, 2011; Wheeler et al., 2018
*Consolida ajacis*	Larkspur	*Meloidogyne* spp.	Wang and McSorley, 2005
*Lavandula angustifolia*	Lavender	*Aphelenchoides ritzemabosi, Meloidogyne hapla*	Kohl, 2011; LaMondia, 1995
*Lilium* sp.	Lily	*Pratylenchus penetrans*	Chitambar et al., 2018
*Eustoma grandiflorum*	Lisianthus	*Meloidogyne* spp.	Wang and McSorley, 2005
*Polystichum adiantiforme*	Leatherleaf fern	*Pratylenchus neglectus*	Rhoades, 1968
*Heliconia*	Lobster Claw	*Helicotylenchus dihystera, Meloidogyne incognita, Pratylenchus* spp.*, Rotylenchulus reniformis*	Bala and Hosein, 1996
*Petunia hybrida*	Petunia	*Aphelenchoides ritzemabosi, Meloidogyne incognita*	Khan et al., 2005; Kohl, 2011; Wheeler et al., 2018
*Papaver rhoeas*	Poppy	*Meloidogyne incognita*	Khan et al., 2005
*Rosa* sp.	Rose	*Aphelenchoides* spp., *Criconemella* spp., *Ditylenchus myceliophagus, Helicotylenchus crenacauda, H. pseudorobustus, H. vulgaris, Meloidogyne hapla, M. incognita, M. javanica, Pratylenchus* spp., *P. neglectus, P. thornei, Rotylenchulus reniformis*	Deimi et al., 2008; Phani et al., 2021; Yamamoto and Toida, 1995
*Ficus* sp.	Rubber plant	*Pratylenchus coffeae*	Pinochet and Duarte, 1986
*Tulipa* sp.	Tulip	*Aphelenchoides ritzemabosi, Ditylenchus dipsaci, Helicotylenchus pseudorobustus, Meloidogyne incognita, Paratrichodorus* spp., *Pratylenchus neglectus, P. thornei, Trichodorus* spp.	Borgohain, 2016; Deimi et al., 2008; Kohl, 2011; Madhavan et al., 2021
*Antirrhinum majus*	Snapdragon	*Aphelenchoides ritzemabosi, Helicotylenchus pseudorobustus, Meloidogyne* spp.	Kohl, 2011; Wang and McSorley, 2005; Wheeler et al., 2018
*Helianthus* sp.	Sunflower	*Aphelenchoides ritzemabosi*; *Meloidogyne incognita*; *Paratylenchus projectus* Jenkins, 1956; *Pratylenchus thornei*	Khan, 2015; Kohl, 2011; Loof, 1975; Rashad et al., 2011
*Zinnia* sp.	Zinnia	*Aphelenchoides besseyi, A. fragariae, A. ritzemabosi, Meloidogyne* spp.	Khan, 2015; Kohl, 2011; Wheeler et al., 2018

*Root-knot nematodes*: The most economically devasting and important PPN is the root-knot nematode, *Meloidogyne* spp., due to its worldwide distribution and host range of over 3,000 plant species Abad et al., 2003). There are over 100 described *Meloidogyne* species, resulting in these nematodes infecting almost every agricultural crop and most weeds Hussey and Janssen, 2002; Elling, 2013). Root-knot nematodes Fig. 1A) are sedentary endoparasites remaining stationary inside the roots of a host plant with the plant growing around them to form galls Taylor and Sasser, 1978). Small galls with usually a single nematode occur on young feeder roots, and larger galls can be a consequence of multiple infections at the same location. In agricultural cultivated fields, there are four major *Meloidogyne* spp. that account for 95% of all root-knot infestations: *M. incognita* (Kofoid and White, 1919) Chitwood, 1949; *M. hapla* Chitwood, 1949; *M. javanica* (Treub, 1885) Chitwood, 1949; and *M. arenaria* (Neal, 1889) Chitwood, 1949 Hussey and Janssen, 2002). Among root-knot nematode species, the northern root-knot nematode, *Meloidogyne hapla*, is the most important perennial ornamental pathogen in the northern United States and Canada LaMondia, 1996), whereas in the southern United States, a variety of tropical root-knot nematode species infect ornamentals Brito et al., 2010).

**Figure 1:**
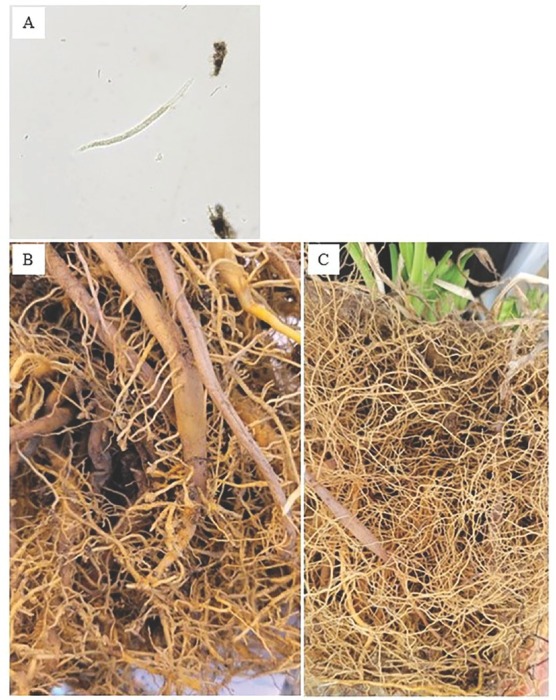
Light micrograph of *Meloidogyne hapla* second-stage juvenile (A) extracted from a daylily field at a commercial nursery in Michigan. Daylily roots were taken from the same field showing galling and stunting due to *M. hapla* infection (B) compared to healthy roots (C).

Symptoms of root-knot nematode infection include galled and stunted roots Fig. 1B), plants that wilt easily and are stunted, and those that have poor vigor and other symptoms common in nutrient deficiencies like chlorosis. Additionally, yield can be dramatically reduced in bare-rooted ornamentals that are grown in the field leading to over 20% yield loss Lindberg et al., 2018). In cut foliage crops, root-knot nematode infection can lead to slower regrowth of leaves and stems Baidoo et al., 2017). Lastly, their characteristic galls can prevent the sale and distribution of ornamental plants, further reducing yield profits. In herbaceous perennial plants that have large, fleshy roots, the northern root-knot nematode galls can easily be inconspicuous and hard to notice leading to the accidental spread of these nematodes in exports. Current management strategies are limited for managing this important pest, and typically consist of hot water dip treatments (Powell and Riedel, 1978; Inserra et al., 1995; Daughtrey and Benson, 2005), where propagules are dipped in hot water for a period of time to kill nematodes, and preplant soil fumigation, which is extremely costly and toxic to the environment. Since 2005, when methyl bromide was banned due to its ozone depleting capabilities, there has been a shift to move away from harmful fumigants to less damaging controls such as biological nematicides and organic amendments Zasada et al., 2010).

*Foliar nematodes*: Foliar nematodes, *Aphelenchoides* spp. Figs. 2A,B), are aerial nematodes that have a wide host range of over 700 plant species, including many ornamental plants like hosta and chrysanthemum Table 1) Handoo, 1998; Jagdale and Grewal, 2006; Kohl, 2011; Mitiku, 2018). The three main species of economic importance to the ornamental industry are *A. fragariae* (Ritzema Bos, 1891) Christie, 1942, *A. ritzemabosi* (Schwartz, 1911) Steiner and Buhrer, 1932, and *A. besseyi* Christie, 1942 Kohl, 2011). Foliar nematodes are primarily endoparasites that feed predominately inside the leaves, but depending on environmental conditions, foliar nematodes can also feed externally on the leaves and flower buds of some plants. These nematodes mostly overwinter in the soil and migrate up the outside of plant stems in films of water to the leaves. They are spread easily through infected plant seeds and are commonly splashed around in water via rain or overhead irrigation from infected plants to plants nearby. Symptoms from foliar nematode feeding are chlorotic, angular lesions on the leaves that run parallel to major leaf veins and can eventually become necrotic if feeding persists Figs. 2C,D). This can lead to leaves having a dry, tattered appearance, and severe infection can kill the whole leaf, causing defoliation Jagdale and Grewal, 2006; Kohl, 2011; Mitiku, 2018; Phani et al., 2021).

**Figure 2:**
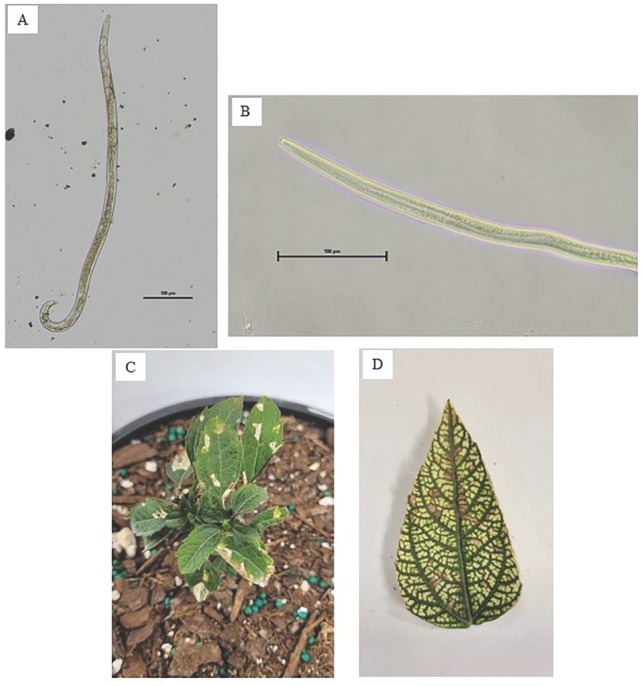
Light micrograph of an adult male (A) and head (B) of *Aphelenchoides* spp. extracted from *Heliopsis* spp. leaves. Angular lesions (C,D) on the leaves of two varieties of *Heliopsis* spp. infected with *Aphelenchoides* spp.

Management of foliar nematodes can be difficult since *Aphelenchoides* spp. can survive in infested dried leaf debris and in dormant plant crowns for many years, and they are easily splashed around Jagdale and Grewal, 2006; El-Saadony et al., 2021). However, there are various methods that can be employed to manage these nematodes. Current management methods include using drip irrigation instead of overhead watering and sanitation of tools, pots, and soil that come in contact with infected plants by heat treating them via baking or steaming Mitiku, 2018). Other treatments include hot water drenches on infected plants, their leaves, and dormant plant materials Jagdale and Grewal, 2004; Kohl, 2011; Mitiku, 2018); the use of humic acid derived from manure Nagachandrabose and Baidoo, 2021); and plant resistance in some plant species Kohl, 2011; Mitiku, 2018; El-Saadony et al., 2021).

*Virus vectoring nematodes*: There are two different types of nematode transmitted viruses based on the viruses’ particle shape: NEPO viruses (nematode polyhedral viruses) and TOBRA viruses (tubular or rod-shaped viruses) Taylor, 1972).

These viruses are transmitted through nematode feeding on plant tissues. NEPO viruses are transmitted by *Xiphinema* (dagger) and *Longidorus* (needle) nematodes; both ectoparasitic nematodes are large and slender. Their host range consists of perennial and woody plants, grapevines, orchids, and small fruits Taylor, 1972; Handoo, 1998). Main NEPO viruses for ornamental plants, such as geraniums, petunia, and tulips, are tomato ringspot virus (ToRSV), tobacco ringspot virus (TRSV), Arabis mosaic virus (ArMV), and tomato black ring (TBRV) virus Engelmann and Hamacher, 2008). While there are numerous NEPO viruses, most of which are species dependent, general symptoms include pronounced ring and mosaic lined patterns, chlorotic flecking, mottling, leaf curling and necrosis, stunted plants, and general plant decline.

TOBRA viruses are transmitted by *Trichodorus* and *Paratrichodorus* species (stubby root nematodes), which are thick nematodes with a curved stylet. Similar to dagger and needle nematodes, they feed externally at the root tip, causing the root tips to swell and become stubby Taylor, 1972). The main viruses are Tobacco mosaic virus (TMV), Tobacco rattle virus (TRV), Pea early-browning virus (PEBV), and Pepper ringspot virus (PepRSV) which can affect plants like petunias, tulips, and lilies Taylor, 1972; Handoo, 1998; Engelmann and Hamacher, 2008; Macfarlane, 2010; Khan, 2015). General symptoms of TOBRA viruses include chlorotic and necrotic spots or strips on leaves, mosaic, mottling, light or dark colored flecks on flower petals, oval lesions, flower malformation, and stunted plants Engelmann and Hamacher, 2008; Madhavan et al., 2021).

Management of these nematodes to prevent virus spread and transmission include using only virus-free planting material, hygienic measures and disinfection of tools and equipment, thermotherapy, destroying virus infected plants to help prevent the spread of inoculum, and regular testing of plant stocks Taylor, 1972; Engelmann and Hamacher, 2008; Adaskaveg and Caprile, 2009; Madhavan et al., 2021). Weed control is especially important since they can serve as alternative hosts for nematodes and can act as virus reservoirs. For example, dandelions, *Taraxacum officinale*, and other broadleaf weeds can harbor viruses, and nematodes can feed on them, obtain the virus, and then transmit it to ornamental plants in the same field Adaskaveg and Caprile, 2009). Fumigants for controlling the nematode vector can be used preplanting as a management option. Additionally, using both nematode and virus resistant cultivars can be a very effective management option. For example, in tulips, there are several TBV-resistant cultivars such as ‘Cantata’, ‘Juan’, ‘Madame Lefeber’, and ‘Princeps’ Madhavan et al., 2021). While these management methods can be effective, there is a need for more research, especially since plant propagules can also harbor viruses leading to their spread.

*Other plant-parasitic nematodes*: There are many other PPNs that can infect and severely damage ornamental plants Table 1). Some of the other main PPNs are *Pratylenchus* spp., or lesion nematode, which is a migratory endoparasite that has a host range of over 400 plant species, including amaryllis Christie and Birchfield, 1958; Nong and Weber, 1965), ferns Rhoades, 1968), and foliage plants such as rubber plants Pinochet and Duarte, 1986). Symptoms of lesion nematode feeding in ornamental plants has been reported to be stunting of roots and shoots, chlorotic foliage, foliage discoloration, wilting, root swelling, brown lesions, destruction of the entire root systems, and severe yield loss Christie and Birchfield, 1958; Rhoades, 1968; Mitiku, 2018). Symptoms can also include necrotic lesions on plant roots which can lead to secondary infections from bacteria and fungi causing disease complexes. *Radopholus similis* (Cobb, 1893) Thorne, 1949, the burrowing nematode, is another migratory endoparasite that is an important pest in anthurium and black pepper Haegeman et al., 2010; Khan, 2015), with over 350 additional hosts. Feeding from *R. similis* in the roots and stems causes root decay and rot, severe plant stunting and chlorosis, and can cause plant dieback and death Borgohain, 2016; Wang et al., 2016). In anthurium rhizomes, *R. similis* can also disrupt the vascular bundles causing necrosis and anatomical alterations in the roots Vovlas et al., 2003), or they can fail to produce any noticeable symptoms in anthurium canes, which are used to plant new fields, leading to the accidental spread of this nematode Sipes and Myers, 2018).

*Rotylenchulus reniformis* Linford and Oliveira, 1940, the reniform nematode, is a semi-endoparasitic nematode that partially penetrates plant roots. These nematodes produce no plant symptoms in some ornamental plants like daylily Inserra et al., 1998), but they can be devasting parasites to crops such as cotton Koenning et al., 2004). *Helicotylenchus* spp., the spiral nematode, is a migratory ectoparasite that has a large host range, but very little research has been conducted on this nematode in ornamental plants, except for *Rotylenchus buxophilus* Golden, 1956, the boxwood spiral nematode, which causes slow decline in boxwood plants Lehman 1984; Eisenback, 2018). Another migratory ectoparasite, *Paratylenchus* spp., or pin nematode, is also commonly found in ornamental plant fields, such as daylily Howland et al., 2022), but to date, very little research has been conducted on this nematode species in ornamental plants except on *P. shenzhenensis* Wang, 2013 in anthurium Wang et al., 2016). Symptoms of *Paratylenchus* spp. infection includes stunting, low quality plants, and decreased yields.

## Plant-Parasitic Nematode Management and Gaps

Plant-parasitic nematodes cost the ornamental plant industry millions of dollars due to their symptoms, yield loss, and plant exportation rejection as described in this review. Current management strategies are not very effective in ornamental production LaMondia, 1996), making management of PPNs in ornamental plants a challenging task. This is especially true due to the loss of effective but environmentally degrading pesticides, propagation and movement of asymptomatic plants, and lack of general knowledge.

**Figure 3:**
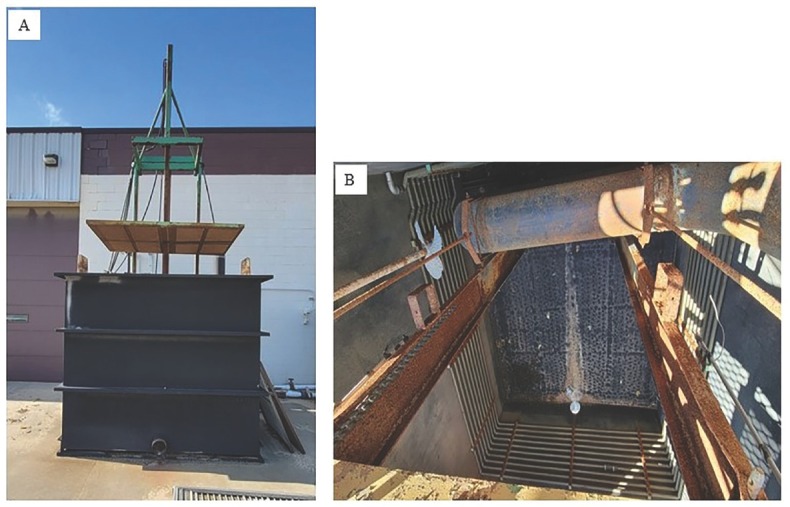
Hot water dipping tank (A) and the interior of the tank (B) at a commercial nursery in Michigan.

For certain ornamental plants, especially those grown exclusively in the field for several years like daylily, iris, and hosta, current management strategies consist of hot water treatments (drenches and dips) and preplant fumigation. Hot water drenches are used to disinfect plants from pests such as insects and PPNs, such as the stem and bulb nematode, *Ditylenchus dipsaci* (Kuhn, 1857) Filipjev, 1936, *A. fragariae*, and *R. similis*. Plant material that is typically treated with hot water are bare-rooted plants, tubers, runners, and dormant plant material, where the material is dipped in a large tank of hot water Figs. 3A,B) then cooled down in secondary water tanks. Hot water dips can kill other endoparasitic nematodes, such as *Meloidogyne* spp., but can have little to no impact on ectoparasitic nematodes that live in the soil and remain outside the roots; planting heat-treated plants in soil already infected with ectoparasitic nematodes will not prevent infection. While effectively killing PPNs, both hot water drenches and dips can cause plant mortality and reduced vigor, germination, and growth in propagules Rhoades, 1964; Jagdale and Grewal, 2004; Tsang et al., 2004; Howland et al., 2022). Dipping the plants in nematicides, instead of just hot water, as shown by Howland et al. (2022), is an alternative management strategy that shows potential. In that trial, daylily plugs were dipped in a fluopyram solution before planting; those plants had significantly higher biomass with moderate control of root-knot nematodes compared to the control and Telone II fumigation. However, testing of nematicide chemicals as a preplant dip or post-harvest treatment in perennials needs further investigation.

The second main management strategy, soil fumigation, can only be applied before planting, and not while plants are in the ground since it is phytotoxic Noling, 2008), thus leading to unsatisfactory control of nematodes after several years of plant growth, especially in the last year of the plant’s production cycle. In addition, most fumigant nematicides are high-risk/high-cost products Zasada et al., 2010), so a management system or a non-phytotoxic product that can be applied throughout the growing cycle is needed. There are several novel nematicides, including fluensulfone, tioxazafen, and fluopyram Phani et al., 2021; Howland et al., 2022) that show promising control, and new chemical products continue to improve while having minimal environmental impact Daughtrey and Benson, 2005).

With an estimated 85,000–99,000 ornamental plant species and their wild relatives worldwide Long et al., 2018), host status screenings of ornamental plants, and their respective numerous varieties, are an important component of PPN management. However, this is an understudied factor in this industry. For example, there are over 83,000 registered cultivars of daylily, yet only a few varieties have actually been tested as hosts to *M. hapla*. Similarly, *R. similis* can contaminate daylily if the plants are grown in the field in conjunction with another host Inserra et al., 1998), but there is no research on *R. similis* infection and impact in daylily. Additional host status screenings of the most economically important ornamental plants are needed to determine resistant, susceptible, and tolerant varieties; this information can be used to determine which varieties can be planted in nematode contaminated fields to help prevent yield loss.

Plant resistance is another under-reported aspect, yet it is a highly effective and inexpensive management strategy to control PPNs in agricultural crops Hajihassani et al., 2018). Some species of plants are genetically resistant to certain PPNs, such as resistance to some *Aphelenchoides* spp. on hosta, but the extent of plant resistance is largely unknown and focuses more on agricultural crops. Protocols to assess resistance have been established in certain plant species, such as on *Aphelenchoides* spp. in hosta Zhen et al., 2012), but this is not available in most ornamental plants.

An important aspect of all pathogen management is integrated pest management (IPM), which is a cornerstone of ornamental plant and nursery crop production Daughtrey and Benson, 2005). Various methods should be used in conjunction with each other to achieve high PPN control, such as cultural practices, chemical control, clean stock programs, sanitation measures, periodic rotation, and plant resistance. Additionally, biocontrols are becoming a more sustainable management strategy that can be applied to ornamental plants. Examples include using nematode-pathogenic fungi that can parasitize PPNs of all life stages such as eggs, juveniles, females, and adults, such as *Purpureocillium lilacinum* Baidoo et al., 2017); these products can even be applied to perennial plants to suppress PPN population levels Crow, 2014). The use of commercial products that contain the bacteria *Bacillus firmus* and *Pasteuria penetrans* have been found to be promising as well Topalović et al., 2020; Phani et al., 2021). However, in a lot of ornamental plants, such as caladium, there is no information available on whether these biological nematicides are effective against PPNs Gu et al., 2022). Efficacy trials on these biocontrol agents should be implemented to determine their effects on PPNs, other soil-borne diseases, and overall soil health.

Environmentally sustainable management strategies of PPNs can be achieved. Pesticides are still the number one management strategy El-Saadony et al., 2021), although alternative nematode management methods including biocontrol, biological nematicides, thermotherapy, and other cultural practices show promising management in the ornamental plant industry Khan et al., 2005; Crow, 2014; Desaeger et al., 2019; Howland et al., 2022).

## Outlook and Future Directions

This review highlights the most important PPNs affecting the ornamental plant industry, but there are many other PPNs whose economic and damage potential remains unknown in this field. For instance, there are only four main *Meloidogyne* species, but there are over 100 described species, and some of these lesser known “minor” species, such as *M. enterolobii* Yang and Eisenback, 1983 and *M. mayaguensis* Rammah and Hirschmann, 1988, are emerging major problems in agriculture and can parasitize ornamental plants Brito et al., 2007; Elling, 2013). Rarely are these PPNs reported on in the ornamental plant industry. Only recently has more research been conducted, with the majority being first reports, host status trials, and tests of new nematicides’ effectiveness in controlling these pests. Surveys, identification, and ecological studies, such as infection behavior and overwintering survival, of some of the less common PPNs can be an important advancement in controlling these pests in the ornamental plant industry.

Another direction ornamental plant management should go in is improving plant breeding techniques to include plant resistance to nematodes. Plant resistance is an efficient tool for controlling nematodes, and the development of resistant varieties can result in reduced yield loss and increased profits in all agricultural industries Boerma and Hussey, 1992). However, with the wide range of PPNs and the main focus being on agricultural crops and not ornamental plants, plant resistance to the most important nematode species described here needs to be studied further, along with the identification of new molecular markers for resistance genes in this field. Similarly, molecular identification techniques need to be improved with potentially new primers developed for less common nematode species. Then, high-yielding, profitable cultivars can be developed to provide growers with consistently effective nematode management.

Since PPNs can easily be spread through asymptotic plants, the development of diagnostic tools to detect and subsequently restrict their movement is crucial to preventing their further distribution. PCR-based diagnostic assays do exist for some nematodes, such as *A. fragariae* McCuiston et al., 2007), *D. dipsaci* Marek et al., 2005), and *R. similis* Krishna and Eapen, 2019). However, these diagnostic assays do not exist for most nematodes and are species-specific; they do not work for intraspecies analysis within a genus. For instance, the PCR-based diagnostic assay developed for *A. fragariae* by McCuiston et al. (2007), does not work on *A. besseyi* or *A. ritzemabosi* since these assays only use species-specific primers. Development of real-time diagnostic assays for more PPNs is highly needed to detect if nematodes are present in plant exports. This will be especially important with climate change increasing the spatial distribution and seasonal variation of PPNs.

In conclusion, limited research has investigated the effect of PPNs in ornamental plants, probably due to the fact that they are not a food crop. However, considering how fast the ornamental industry is growing and the increasing demand for ornamental flowers and plants, more research needs to be conducted on finding new management options, increasing plant resistance, and better understanding nematode epidemiology. Controlling these nematodes can help prevent their spread through exports and can help reduce yield loss worldwide.
